# Effects of HCl concentration and immersion time on physicochemical properties, BMP-2 detection, degradation, and osteoblast response of human demineralized tooth matrix

**DOI:** 10.2340/biid.v13.45645

**Published:** 2026-03-27

**Authors:** Anupong Jeerachaipansakul, Narit Leepong, Srisurang Suttapreyasri

**Affiliations:** Department of Oral and Maxillofacial Surgery, Faculty of Dentistry, Prince of Songkla University, Hat Yai, Songkhla, Thailand

**Keywords:** human demineralized tooth matrix, tooth-derived graft, hydrochloric acid, demineralization parameters, degradation, BMP-2, osteoblast response

## Abstract

**Objective:**

Human demineralized tooth matrix (hDTM) is a promising tooth-derived graft material; however, demineralization protocols vary widely and may alter matrix structure, degradation behavior, and growth factor detectability. This study investigates the combined effects of hydrochloric acid (HCl) concentration (0.5 vs 1.0M) and immersion time (10 vs 20 min) on the physicochemical characteristics, Bone Morphogenetic Protein-2 (BMP-2) detection, degradation behavior and osteoblast response of hDTM.

**Methods:**

Caries-free permanent teeth (particle size 500–1000 μm) were demineralized using four HCl protocols: 0.5M/10 min, 0.5M/20 min, 1M/10 min, and 1M/20 min. Morphology, crystallinity, and surface features were characterized by scanning electron microscopy (SEM), X-ray diffraction, X-ray fluorescence, and Brunauer-Emmett-Teller analysis. Total protein and BMP-2 were quantified using Bradford and enzyme-linked immunosorbent assay (ELISA) assays. In-vitro degradation was monitored for 60 days in simulated body fluid. MC3T3-E1 osteoblast adhesion and proliferation were evaluated using the SEM and PrestoBlue^®^ assay. Two-way analysis of variance was performed to assess main and interaction effects of concentration and time.

**Results:**

Both HCl concentration and immersion time significantly influenced hDTM properties. Lower concentration enlarged dentinal tubules and collagen exposure, whereas higher concentration produced surface microcracking and reduced collagen exposure. Higher HCl concentration increased degradation (1M/20 min: 23.76 ± 3.23% vs. 0.5M/20 min: 9.02 ± 0.63%, p < 0.01), while longer immersion increased ELISA-detected BMP-2 levels (1M/20 min: 11.8 ± 1.9 ng/g; 0.5M/20 min: 5.3 ± 1.6 ng/g vs. control: 0.1 ng/g, *p* < 0.001). Significant independent and interactive effects of HCl concentration and immersion time were observed (*p* < 0.001). Among the test conditions, a 0.5M/20 min protocol demonstrated balanced collagen preservation, controlled degradation, and favorable osteoblast proliferation.

**Conclusion:**

HCl concentration and immersion time independently and interactively modulate hDTM properties. Within the test conditions, 0.5M HCl for 20 minutes provides a practical solution and balances collagen preservation, controlled degradation, and osteoblast proliferation, supporting its use as a feasible approach for fabricating tooth-derived bone graft materials.

## GRAPHICAL ABSTRACT

**Figure:**
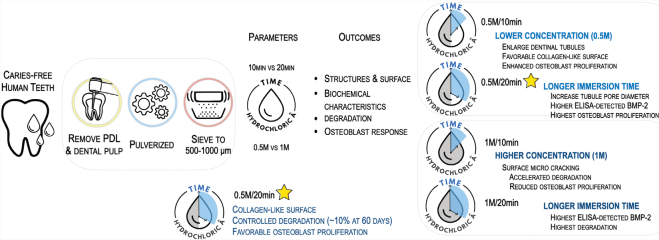

KEY MESSAGES

- Hydrochloric acid (HCl) concentration and immersion time influenced the physicochemical characteristics, degradation behavior, Bone Morphogenetic Protein-2 (BMP-2) levels detected by enzyme-linked immunosorbent assay (ELISA), and osteoblast response of human demineralized tooth matrix (hDTM).
- Lower HCl concentration preserved collagen-like surface features and supported favorable osteoblast proliferation, whereas higher HCl concentration produced surface microcracking and accelerated degradation.
- Among the tested protocols, 0.5M HCl for 20 minutes provided a favorable balance of dentinal tubule exposure, collagen-like surface feature, controlled degradation (10% over 60 days), and enhanced osteoblast proliferation.

## Introduction

Alveolar bone defects are frequently encountered in oral and maxillofacial surgery and implant dentistry as a consequence of tooth extraction, trauma, periodontal disease, and infection. Reconstruction of deficient ridge volume is often required to achieve functional and esthetic implant-supported rehabilitation. Although autogenous bone graft remains the clinical gold standard due to its osteogenic, osteoinductive, and osteoconductive properties, its use is limited by donor-site morbidity, increased operative time, limited available volume, and unpredictable resorption. In addition, allografts and xenografts may present limitations related to cost, variability in biological performance, slow remodeling, or patient acceptance, while synthetic substitutes may lack the biological cues necessary for robust bone regeneration.

Human teeth represent a promising autogenous biomaterial for alveolar bone regeneration due to their compositional similarity to bone tissue [1]. The inorganic component consists primarily of hydroxyapatite (HA) [2] while the organic matrix of dentin predominantly comprises collagenous proteins and a smaller proportion of non-collagenous proteins including growth factors such as bone morphogenetic proteins (BMPs), phosphoproteins, osteocalcin, proteoglycans, and dentin sialophosphoproteins [3–5].

The osteoinductive potential of demineralized tooth-derived bone graft was first verified in 1970 by Huggins and Urist [6]. Since then, demineralized teeth have been recognized as both osteoinductive and osteoconductive grafting materials. However, the preparation of human demineralized tooth matrix (hDTM) involves multiple chemical processes that can substantially influence the physicochemical properties, degradation behavior, biocompatibility, and osteogenic potential of the resulting material.

Demineralization, typically achieved through acid or chelating agents, partially removes the mineral content and exposes a collagen-rich framework while maintaining aspects of the inorganic structure. This process may affect surface morphology, porosity, and the detectability of matrix-associated proteins including BMP-2 [7, 8]. Different agents and protocols have been employed, including hydrochloric acid (HCl; 0.1 N to 2.4M [9–11]), ethylenediaminetetraacetic acid (EDTA; 4–15% [11, 12]), and nitric acid (5–10% [13, 14]), with exposure times ranging from minutes to extended periods depending on whether whole teeth or particles are treated. Among these, HCl is commonly used due to its efficiency and rapid mineral removal capability, and clinically practical protocols have ranged from low concentration with long exposure time to higher concentration and short exposure times, depending on the intended degree of demineralization.

Despite increasing interest in hDTM, the literatures reported considerable heterogeneity in demineralized dentin/tooth matrix preparation and outcomes [15–17]. Recent systematic reviews and clinical studies have highlighted wide variations in demineralized protocols, particularly acid type, concentration, and immersion time, which can produce matrices with different surface microstructures, mineral removal levels, and biological responses [18, 19]. Experimental comparisons also demonstrate that chemical treatment conditions generate distinct microstructural features, including differences in surface morphology, dentinal tubule exposure, and structural disruption such as microcracking, which may contribute to divergent cell–material interactions reported across studies [20]. Furthermore, even within HCl-based approaches, exposure time has been reported to vary substantially (e.g. 10–90 minutes in some protocols), suggesting that incomplete standardization may underlie the variability in physicochemical properties and biological performance [21].

Therefore, establishing practical and reproducible demineralization conditions is essential to improve consistency and facilitate translation of tooth-derived graft materials. In this study, we investigated the combined effects of HCl concentration (0.5M vs 1.0M) and immersion time (10 vs 20 minutes) on the physicochemical characteristics, degradation behavior, enzyme-linked immunosorbent assay (ELISA)-detected BMP-2 levels, and osteoblast response of hDTM. This factorial comparison aimed to identify a suitable processing protocol among the tested parameter combinations for fabrication of tooth-derived graft materials.

## Materials and methods

### Ethical considerations and study design

This study was conducted following ethical approval by the Human Research Ethics Committee, Faculty of Dentistry, Prince of Songkla University, Thailand (Protocol number: MOE 0521.1.03/1737). Written informed consent was obtained from all participants prior to tooth extraction, including authorization for the use of extracted teeth for research purposes.

### Sample size and replicates

Sample sizes were determined based on feasibility, pilot variance estimates, and established practice in biomaterials characterization. To improve processing reproducibility and reduce tooth-to-tooth variability, each demineralization condition was prepared in 2–3 independent processing batches (independent preparation runs through the full demineralization workflow). Materials from the independent batches within the same condition were subsequently pooled and homogenized to obtain a sufficient mass for multi-instrument physicochemical characterization and to reduce variability associated with tooth-to-tooth differences. For physicochemical analysis, *n* = 3–5 per group were selected following standard materials characterization protocols. For biological assays, *n* = 5 independent culture replicates per group per time point were used. The sample size was determined a priori to detect a 20% difference in cell proliferation with 80% power and α = 0.05, based on preliminary data variance.

### Tooth collection and preprocessing

Caries-free permanent third molars and premolars extracted for orthodontic indications were collected from the Oral and Maxillofacial Surgery Clinic, Dental Hospital, Faculty of Dentistry, Prince of Songkla University. Teeth were excluded if they presented any pathology/anomaly affecting the tooth structure. After extraction, teeth were thoroughly rinsed under running water to remove blood and debris and then separated into crown and root portions. Adherent soft tissues, including periodontal ligament remnants, were removed using a high‐speed diamond bur. Pulp tissue was mechanically removed by sterile endodontic files, followed by repeated rinsing with double-distilled water to minimize residual soft tissue components.

The cleaned teeth were pulverized into small particles by mixer mill machines (Mixer Mill M301, Retsch GmbH, Haan, Germany). Particles were sieved using sieves with 500 μm and 1000 μm apertures (Endecotts, London, UK). The fraction between 500 and 1000 μm was collected for subsequent demineralization procedures.

### Demineralization protocols

Tooth particles (500–1000 μm) were subjected to four demineralization protocols using HCl (Himedia, Maharashtra, India): (1) 0.5M HCl for 10 minutes (0.5M/10 min), (2) 0.5M HCl for 20 minutes (0.5M/20 min), (3) 1M HCl for 10 minutes (1M/10 min), and (4) 1M HCl for 20 minutes (1M/20 min).

The ratio of tooth particles to HCl solution was 1:10 (w/v). Demineralization was performed at room temperature (25 ± 2°C) with continuous stirring. Following demineralization, samples were thoroughly washed with double-distilled water until neutral pH was achieved, and freeze-dried for 24 hours (Labogene ApS, Germany). Untreated human mineralized tooth matrix (0M/0 min) served as the control group.

Each protocol was prepared in 2–3 independent processing batches, and materials were pooled and homogenized prior to physicochemical characterization.

### Physicochemical characterization

Scanning electron microscopy (SEM) was performed using a scanning electron microscope (FEI Quanta 400, Thermo Fisher Scientific, Brno, Czech Republic)) at an accelerating voltage of 15 kV. Samples were dried, sputter-coated with gold thin film. Electron micrographs were obtained at ×500, ×7000, and ×30,000 magnification (*n* = 3 per group).

X-ray Diffraction (XRD) Analysis: The phase composition and crystallinity percentage of the tooth matrix were characterized by X-ray diffractometer (Empyrean, PANalytical, Netherlands) with Cu-Kα radiation (λ = 1.54 Å) operating at 40 kV and 30 mA with a scanning range (2θ):5–90°, step size (2θ): 0.026°, and time/step: 70.125 seconds. The crystallinity was calculated using a crystallinity index calculated from the XRD pattern according to the following equation:


$$\%\text{crystallinity}=\frac{\text{A}\;\left(\text{peak}\right)}{\text{A}\;\left(\text{total}\right)}\times 100$$

Where A(peak) is the sum of net intensity peak (cts) and A(total) is the sum of net intensity total (cts) (*n* = 3 per group).

X-ray Fluorescence (XRF) Analysis: The elemental composition was determined by X-ray fluorescence spectrometer (XRF, PW 2400, PHILIPS, The Netherlands). The stoichiometric Ca/P ratio was calculated using the following formula [22] (*n* = 3 per group):


$$\frac{\text{Ca}\;\left(\text{mol}\right)}{\text{p}\;\left(\text{mol}\right)}=\frac{\text{Ca}\;\left(\text{Wt\%}\right)}{40.08\;\left(\text{g}/\text{mol}\right)}/\frac{\text{p}\;\left(\text{Wt\%}\right)}{30.97\left(\text{g}/\text{mol}\right)}$$

Brunauer-Emmett-Teller (BET) Analysis: The nitrogen adsorption–desorption experiment was performed to determine specific surface area and average pore diameter (BET, Micromeritics ASAP2460, Micromeritics Instrument Corp., Atlanta, GA, USA) (*n* = 3 per group).

### Protein extraction and BMP-2 quantification

Protein extraction was performed using the guanidine HCl/EDTA method [23]. A total of 100 mg of tooth matrix was placed in 5 mL tubes containing 1.9 mL of 4M guanidine HCl and 50 mM EDTA in 50 mL Tris (pH 7.4) with protease inhibitors. The samples were agitated at 4°C for 24 hours, and supernatants were collected.

The total protein concentration was determined using the Bradford Protein Assay Kit (Pierce Coomassie Plus; Thermo Fisher Scientific, Waltham, MA, USA) according to the manufacturer’s instructions. Measurements were performed in triplicate (*n* = 4 per group).

BMP-2 levels were quantified using a commercially available ELISA kit (Quantikine, BMP-2 Immunoassay, R&D Systems, Minneapolis, MN, USA) according to the manufacturer’s instructions. The BMP-2 concentration was expressed as ng per gram of dry weight. Each sample was analyzed in triplicate (*n* = 4 per group).

### Degradation analysis

In vitro degradation was assessed in simulated body fluid (SBF), prepared according to Kokubo’s protocol [24]. Samples (*n* = 5 per group per time point) were immersed in SBF solution (pH 7.4) at 37°C with gentle shaking. At predetermined time points (0, 1, 3, 5, 7, 14, 21, 30, and 60 days), samples were retrieved, gently rinsed with distilled water, freeze-dried, and weighed. The degradation percentage was calculated as:


$$\text{Degradation}\;\text{rate}\;\left(\%\right)=\frac{\left(\text{Wd0}-\text{Wdt}\right)}{\text{Wd0}}\times 100$$

Where Wd0 is the initial dry weight and Wdt is the dry weight at time t. SBF was refreshed every 3 days to maintain pH and ion concentrations.

### Cell culture and biocompatibility assessment

#### Scaffold preparation

The scaffold structures were created using polyvinyl alcohol (PVA, Mw 145,000 Da; Merck, Germany) at the base for biological assay. The tooth matrix scaffolds were synthesized according to a previously established protocol with modifications [2]. Briefly, 200 mg of tooth matrix was added to 100 μL of 5% wt/v of PVA solutions and transferred into containers (diameter 10 mm, height 3 mm). All samples were freeze-dried for 3 days before sterilization with hydrogen peroxide gas and were maintained under sterile conditions before use.

#### Cell culture and cell scaffold constructs

MC3T3-E1 pre-osteoblast cells (American Type Culture Collection; ATCC, Manassas, VA, USA) were cultured in basal medium consisting of α-MEM (Gibco, Invitrogen, Carlsbad, CA, USA) supplemented with 10% fetal bovine serum, 0.1% fungizone, and 1% penicillin–streptomycin at 37°C with 5% CO2 until reaching confluence. Subculturing was performed using trypsin/EDTA. Cells at passages 4–6 were used for all experiments. Before cell seeding, the scaffolds were placed in a 48-well plate and soaked with basal medium at 37°C for 24 hours.

MC3T3-E1 pre-osteoblast cells (5 × 10^4^ cells) were seeded onto each scaffold and incubated at 37°C for 2 hours. The cell-scaffold constructs were cultured in 2 mL of basal medium with a medium replacement every 2 days.

#### Cell adhesion assay

The cell adhesion and morphology of MC3T3-E1 cells on the scaffolds were analyzed using SEM (Quanta 400, FEI, Oregon, USA). Cells were cultured for 1 or 7 days as described earlier, rinsed twice with PBS, and fixed in 10% formaldehyde for 1 hour at 25°C. Then, samples were dried and sputter-coated with gold thin film. The electron micrographs were obtained at ×300, ×1500, and ×3000 magnification (*n* = 3 per group per time point). For each group, SEM images were collected from multiple randomly selected fields of view, and representative images are presented.

#### Cell proliferation assay

Proliferation of MC3T3-E1 pre-osteoblast cells on the scaffolds was assessed using PrestoBlue^®^ cell viability reagent (Invitrogen, Thermo Fisher Scientific, Waltham, MA, USA), a resazurin-based, membrane-permeable reagent that upon reduction forms resorufin, a product that can be detected spectrophotometrically. At each time point (1, 3, 7, 14, and 21 days of culture), the cell-scaffold constructs (*n* = 5 per group per time point) were washed with phosphate**-**buffered saline (PBS), and then PrestoBlue^®^ reagent (100 μL) was added to 900 μL of culture medium directly over the cells and incubated at 37°C for 60 minutes according to the manufacturer’s protocols. Aliquots of 200 μL were transferred to a 96-well plate, and the optical density was measured at 570 nm using a microplate reader (Thermo Scientific, Wyman Street, Waltham, MA, USA).

### Statistical analysis

Data are presented as mean ± standard deviation (SD). Statistical analyses were performed using IBM SPSS Statistics Standard 29.0.0.0 (IBM Corp., Armonk, NY, USA). Shapiro–Wilk test was used to test distribution, and all data were subject to a normal distribution. One-way analysis of variance (ANOVA) followed by Tukey’s post-hoc test was used for multiple comparisons. Statistical significance was set at *p* < 0.05. Effect sizes (Cohen’s *d* or eta-squared) were calculated where appropriate to assess practical significance. In addition, two-way ANOVA was performed to evaluate the combined effects of HCl concentration (0.5M, 1M) and immersion time (10 and 20 minutes) on selected parameters including total protein, BMP‑2 content, and degradation rate. When significant effects were detected, Tukey’s post-hoc test was applied. Partial η² was reported as the effect size, and significance was set at *p* < 0.05.

## Results

### Physicochemical characteristics

#### Morphological analysis

**Figure 1:**
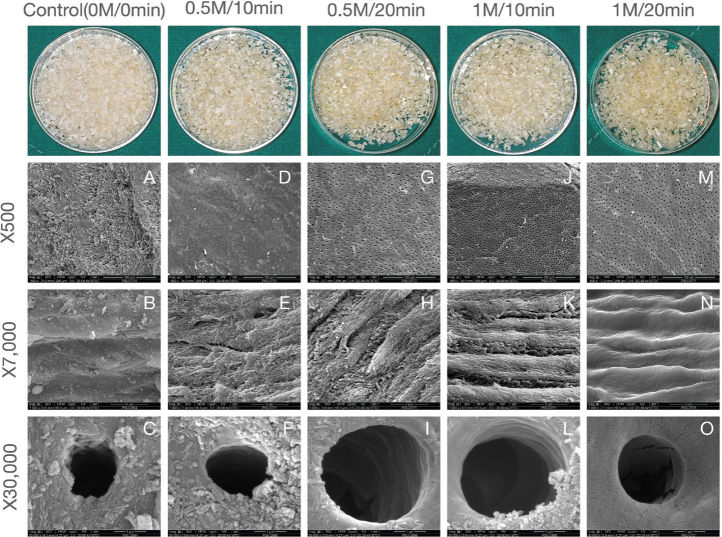
Macroscopic and scanning electron microscopy (SEM) analysis of tooth matrix particles under different demineralization conditions (n = 3 per group). The top row shows the gross morphology of particles in each treatment group. SEM images at ×500, ×7,000, and ×30,000 magnifications illustrate progressive enlargement of dentinal tubules and exposure of collagen fibrils with 0.5 M HCl treatment, while the 1 M HCl groups exhibit surface microcracks and smoother collagen layers, suggesting excessive demineralization.

Macroscopic examination, as shown in Figure 1 (top row), revealed that mineralized tooth particles (control; 0M/0 min) appeared white and opaque, while increasing HCl concentration and immersion time resulted in particles that became progressively more yellow and transparent. All groups maintained hard consistency.

SEM analysis revealed that demineralization parameters significantly influenced hDTM microstructure (Figure 1). The control (0M/0 min; Figure 1A–C) group exhibited irregular surfaces with small dentinal tubules (0.5–1 μm). Treatment with 0.5M HCl (0.5M) enlarged dentinal tubules (Figure 1D-F, 1G-I) to 2–3 μm and enhanced collagen fibril exposure (Figure 1D–F). In contrast, 1M HCl treatment (1M) produced similar tubule enlargement but resulted in limited collagen exposure and visible surface cracking (Figure 1J–L, 1M–O).

**Figure 2:**
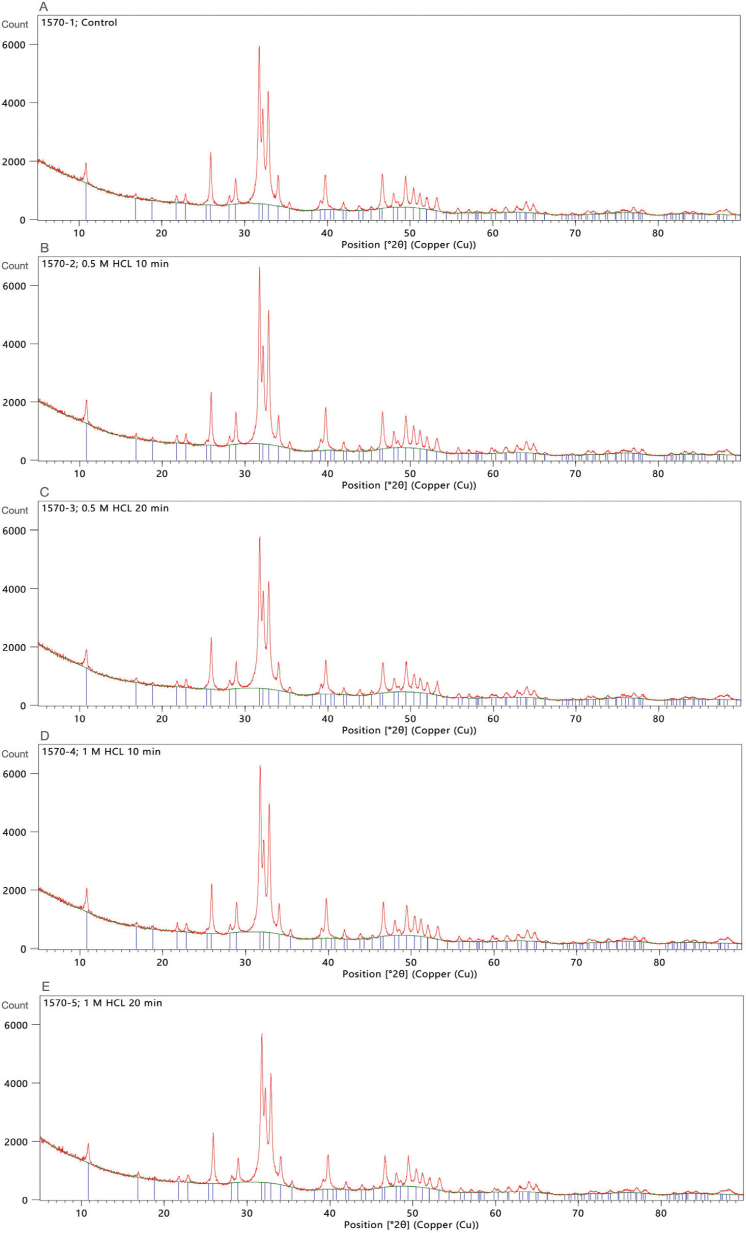
X-ray diffraction (XRD) patterns of tooth matrix particles under different demineralization conditions (n = 3 per group). (A) Control (0M/0min), (B) 0.5M/10min, (C) 0.5M/20min, (D) 1M/10min, (E) 1M/20min. All samples show characteristic hydroxyapatite peaks without secondary phase formation..

XRD patterns (Figure 2) indicated the presence of only the HA phase in all groups without secondary phase transformation. Crystallinity decreased significantly with increasing demineralization intensity: 1M/20 min (54.06 ± 0.12%, *p* < 0.001, Cohen’s *d* = 2.8 vs control), 0.5M/20 min (56.42 ± 0.34%, *p* < 0.001, *d* = 2.1), and 1M/10 min (61.2 ± 0.51%, *p* = 0.004, *d* = 1.2) compared to 0M/0 min (63.20 ± 0.33%) and 0.5M/10 min (62.44 ± 0.24%, *p* > 0.05 vs. control).

#### Elemental composition

XRF analysis confirmed decreased calcium (Ca) and phosphorus (P) content ranging from 8 to 11 wt% and 2 to 4 wt%, respectively, with the most pronounced reduction in 1M/20 min (Ca: 3.43wt%, P: 1.07wt%), compared with the control (Ca: 15.6wt%, P: 8.08wt%, *p* < 0.001). The Ca/P molar ratio increased from 1.49 (0M/0 min) to 1.968 (0.5M/10 min), 2.352 (1M/10 min), 2.391 (0.5M/20 min), and 2.477 (1M/20 min).

#### Surface area and pore diameter analysis

**Figure 3:**
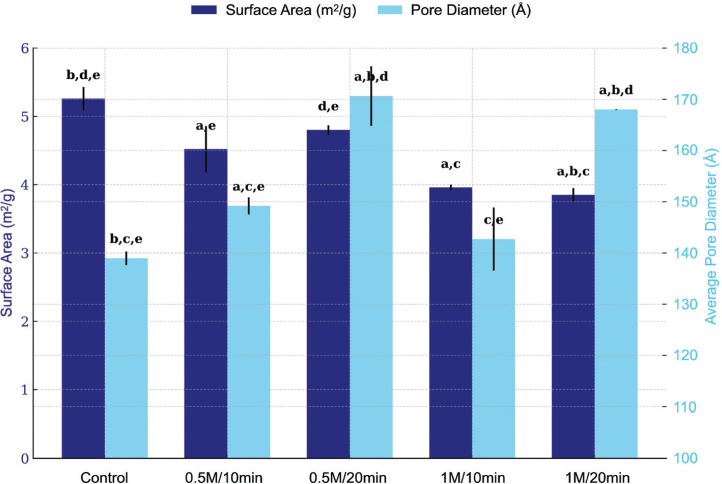
Surface area and average pore diameter of demineralized tooth particles determined by BET analysis (n = 3 per group). Statistical significance (p < 0.05) was determined using one-way ANOVA followed by Tukey’s post hoc test. Different letters above lines indicate significant pairwise differences between groups: a, significantly different from the control group (0M/0min); b, from 0.5M/10min; c, from 0.5M/20min; d, from 1M/10min; e, from 1M/20min..

BET analysis (Figure 3) revealed that demineralization significantly altered the surface characteristics compared with the non-demineralized control.

Demineralization significantly reduced surface area in a concentration-dependent manner. The control group (5.26 ± 0.17 m²/g) exhibited significantly higher values than 0.5M/10 min (4.52 ± 0.34 m²/g; *p* < 0.001), 1M/10 min (3.96 ± 0.04 m²/g; *p* < 0.001), and 1M/20 min (3.85 ± 0.10 m²/g; *p* < 0.001) but was similar to 0.5M/20 min (4.80 ± 0.07 m²/g; *p* = 0.001). Surface areas remained stable at 1M regardless of immersion time (*p* = 0.313), suggesting a saturation effect.

Pore diameter expansion was primarily time-dependent. The 20-minute treatments produced substantially larger pores (0.5M/20 min: 170.64 ± 5.82 Å; 1M/20 min: 168.00 ± 0.07 Å) compared to the control (138.98 ± 1.31 Å; both *p* < 0.001), and their similarity (*p* = 0.269) indicates convergent pore modification independent of concentration. In contrast, 1M/10 min exhibited no significant difference from control (142.71 ± 6.16 Å; *p* = 0.129), whereas 0.5M/10 min (149.20 ± 1.66 Å) demonstrated significant expansion (*p* = 0.001). At 10 minutes, higher concentration produced less pore expansion (*p* = 0.016), demonstrating that treatment duration is the primary determinant of pore diameter modification.

BET analysis demonstrated that surface area decreased primarily with higher concentration, while average pore diameter increased mainly with longer immersion. Two‑way ANOVA indicated a significant concentration effect on surface area and a strong time effect on pore diameter; the interaction term was negligible.

### Protein content and BMP-2 quantification

#### Total protein content

All demineralization protocols increased total protein content compared with the control (0.97 ± 0.08 μg/mL) as shown in Table 1. The greatest protein release occurred in the 1M/20 min group (3.53 ± 0.24 μg/mL, *p* < 0.001), followed by 0.5M/20 min (1.88 ± 0.11 μg/mL, *p* < 0.001) and 1M/10 min (1.84 ± 0.10 μg/mL, *p* < 0.001). In contrast, the 0.5M/10 min group (1.17 ± 0.31 μg/ml, *p* = 0.174) exhibited no significant difference from the control. No significant difference was observed between the 0.5M/20 min and 1M/10 min groups (*p* = 0.994). These results indicate that both acid concentration and immersion time influenced total protein extraction, with prolonged exposure having a greater effect.

**Table 1:** Total protein and BMP-2 content of samples following different HCl demineralization protocols.

Demineralized protocol	Total protein (μg/mL	BMP-2 (ng/g)
Control;0M/0 min	0.97 ± 0.08^c,d,e^	0.03 ± 0.05^c,d,e^
0.5M/10 min	1.17 ± 0.31^c,d,e^	1.12 ± 0.07^c,e^
0.5M/20 min	1.88 ± 0.11^a,b,e^	5.28 ± 1.55^a,b,e^
1M/10 min	1.84 ± 0.10^a,b,e^	3.10 ± 2.37^a,e^
1M/20 min	3.53 ± 0.24^a,b,c,d^	11.81 ± 1.93^a,b,c,d^
*P*-value	< 0.0001	< 0.0001

Statistical significance (*p* < 0.05) was determined using one-way ANOVA followed by Tukey’s post hoc test. Different letters above lines indicate significant pairwise differences between groups:

a Significantly different from the control group (0M/0 min);

b From 0.5M/10 min;

c From 0.5M/20 min;

d From 1M/10 min;

e From 1M/20 min.

BMP-2: Bone Morphogenetic Protein-2; ANOVA: analysis of variance; HCl: hydrochloric acid.

#### BMP-2 content

Similarly, extended demineralization time (20 min vs. 10 min) markedly increased the BMP-2 level detected by ELISA at both HCl concentrations (Table 1). The highest BMP-2 content was found in the 1M/20 min group (11.81 ± 1.93 ng/g), followed by 0.5M/20 min (5.28 ± 1.55 ng/g), both significantly higher than the non-demineralized control (0.03 ± 0.05 ng/g; *p* < 0.001 and *p* = 0.002, respectively). The 1M/10 min group (3.10 ± 2.37 ng/g) also exhibited significantly higher BMP-2 levels than the control (*p* = 0.015), while 0.5M/10 min (1.12 ± 0.07 ng/g) did not differ significantly (*p* = 0.174). No significant difference was detected between the 0.5M/20 min and 1M/10 min groups (*p* = 0.994). Collectively, these findings suggest that both increasing acid concentration and extending demineralization time enhance BMP-2 levels detected by ELISA, with the most pronounced effect observed at 1M HCl for 20 minutes.

Two-way ANOVA confirmed that both acid concentration and immersion time significantly affected total protein and BMP‑2 contents (*p* < 0.001) (Table 2). For total protein, significant main effects were observed for concentration (*F*(1,15) = 149.92, *p* < 0.001, η² = 0.91) and time (*F*(1,15) = 158.75, *p* < 0.001, η² = 0.91), with a strong interaction (*F*(1,15) = 26.25, *p* < 0.001, η² = 0.64). BMP‑2 content exhibited similar patterns, with significant effects of concentration (*F*(1,15) = 30.77, *p* < 0.001, η² = 0.67) and time (*F*(1,15) = 70.51, *p* < 0.001, η² = 0.83), and a moderate interaction (*F*(1,15) = 8.79, *p* = 0.010, η² = 0.37).

**Table 2:** Two‑way ANOVA results for effects of HCl concentration and immersion time on hDTM properties.

Dependent variable	Source of variation	*F*(df₁, df₂)	*P*-value	Partial η²	Interpretation
Total protein	Concentration	149.92 (1, 15)	< 0.001	0.909	Strong effect – protein ↑ with acid
	Time	158.75 (1, 15)	< 0.001	0.914	Strong effect – protein ↑ with longer time
	Interaction	26.25 (1, 15)	< 0.001	0.636	Significant synergy
BMP‑2	Concentration	30.77 (1, 15)	< 0.001	0.672	Strong effect – BMP‑2 ↑ with acid
	Time	70.51 (1, 15)	< 0.001	0.825	Strong effect – BMP‑2 ↑ with time
	Interaction	8.79 (1, 15)	0.010	0.369	Moderate synergy
Degradation (day 60)	Concentration	163.98 (1, 220)	< 0.001	0.427	Strong effect – degradation ↑ with acid
	Time	13.09 (1, 220)	< 0.001	0.056	Moderate effect – degradation ↑ with time
	Interaction	0.54 (1, 220)	0.463	0.002	Not significant

ANOVA: analysis of variance; BMP-2: Bone Morphogenetic Protein-2; hDTM: human demineralized tooth matrix; HCl: hydrochloric acid; partial η² Partial η² reported as effect size; significance set at *p* < 0.05.

#### Degradation behavior

**Figure 4:**
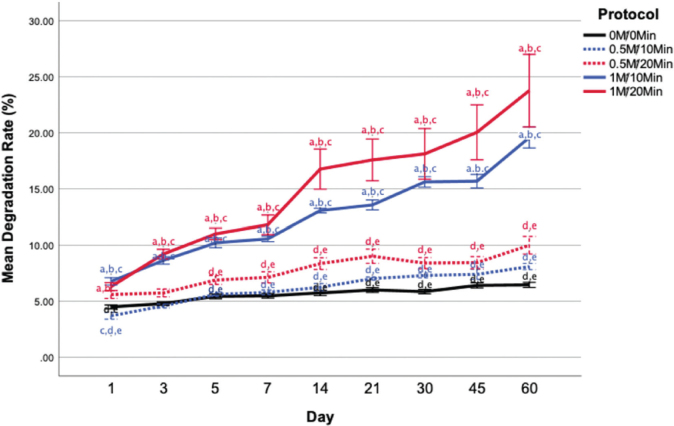
In vitro degradation behavior of tooth matrix in simulated body fluid (SBF) over 60 days (n = 5 per group per time point). Mean degradation rate (%) is shown for different demineralization conditions. Statistical significance (p < 0.05) was determined using one-way ANOVA followed by Tukey’s post hoc test. Different letters above lines indicate significant pairwise differences between groups: a, significantly different from the control group (0M/0min); b, from 0.5M/10min; c, from 0.5M/20min; d, from 1M/10min; e, from 1M/20min..

The degradation rate of the tooth matrix increased progressively with both acid concentration and immersion time (Figure 4). All demineralized groups exhibited higher degradation rates than the control throughout the 60-day period. During the early phase (days 1–7), both 1M HCl groups demonstrated significantly greater degradation compared with the control and 0.5M groups (*p* < 0.001). This trend persisted through days 14–60, with 1M HCl treatments maintaining consistently higher degradation rates across all time points.

At day 60, the 1M/20 min group exhibited the highest degradation rate (23.76 ± 3.23%), followed by 1M/10 min (19.57 ± 0.93%). Both 1M groups exhibited significantly greater degradation than 0.5M/10 min (8.09 ± 0.28%; *p* < 0.001), 0.5M/20 min (9.02 ± 0.63%; *p* < 0.001), and the control (6.46 ± 0.23%; *p* < 0.001).

Two-way ANOVA revealed significant main effects of acid concentration (*F*(1, 220) = 163.98, *p* < 0.001, η² = 0.43) and immersion time (*F*(1, 220) = 13.09, *p* < 0.001, η² = 0.06) on degradation rate, with no significant interaction between factors (*p* = 0.46). These findings, along with effects on total protein content and BMP-2 levels detected by ELISA, are summarized in Table 2.

### Cell biocompatibility assessment

#### Cell adhesion

**Figure 5:**
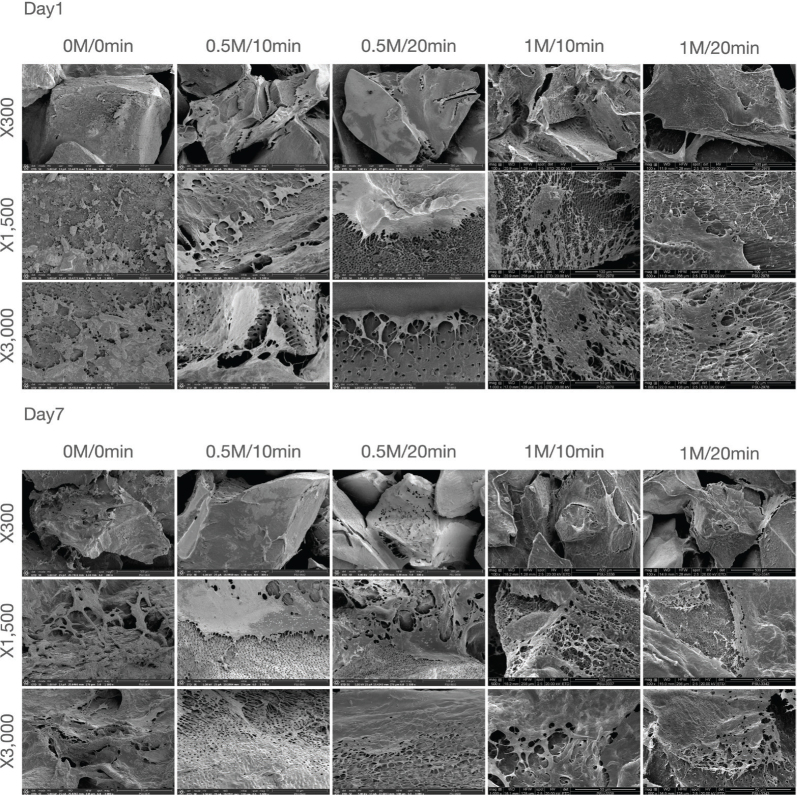
Cell adhesion and morphology on human demineralized tooth matrix (hDTM) scaffolds analyzed by scanning electron microscopy (SEM) at Day 1 and Day 7 (n = 3 per group per time point). Representative SEM images of samples prepared under different demineralization protocols (0M/0min, 0.5M/10min, 0.5M/20min, 1M/10min, and 1M/20min) are presented at ×300, ×1,500, and ×3,000 magnifications. Images at different magnifications were acquired from different fields of view of the same specimen/group and therefore are not shown as matched zoom-in regions. Scale bars are shown in each image. On Day 1, demineralized groups showed enhanced cell spreading and attachment versus the control. By Day 7, increased cell density, pseudopodia extension, and multilayer formation indicated improved surface bioactivity after demineralization..

SEM images of cell morphology and attachment on hDTM scaffolds are shown in Figure 5. On day 1, MC3T3-E1 cells with a spreading morphology were observed in all demineralized protocol groups. In contrast, cells in the control group were deposited and aggregated on the scaffold surface, with most exhibiting a rounded morphology. By day 7, the cell numbers had increased in all protocol groups, with cells spreading and extending pseudopodia, growing in multilayers, and forming dense cell sheets on the scaffold surface.

#### Cell proliferation

**Figure 6:**
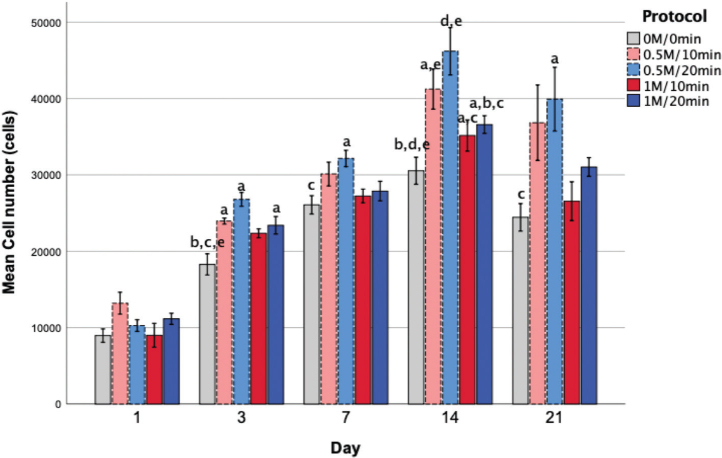
Proliferation of MC3T3-E1 cells on human demineralized tooth matrix (hDTM) scaffolds assessed by PrestoBlue assay (n = 5 per group per time point). Cell proliferation increased over time in all groups, peaking on Day 14; the 0.5M/20min group showed the highest cell numbers. Statistical significance (p < 0.05) was determined using one-way ANOVA followed by Tukey’s post hoc test. Different letters above lines indicate significant pairwise differences between groups: a, significantly different from the control group (0M/0min); b, from 0.5M/10min; c, from 0.5M/20min; d, from 1M/10min; e, from 1M/20min..

Cell proliferation, as shown in Figure 6, increased gradually in all groups, reaching peak levels on day 14. The 0.5M/20 min group exhibited the highest cell numbers compared to other groups, with a significant difference from the control from day 3 to day 21. Notably, lower HCl concentration protocols (0.5M/10 min and 0.5M/20 min) demonstrated superior cell proliferation compared to higher HCl concentration protocols (1M/10 min and 1M/20 min).

## Discussion

The demineralization process using acid extraction represents a critical step influencing the exposure of organic matrix components, including collagenous and non-collagenous proteins in tooth-derived bone graft substitutes. This study evaluated how HCl concentration and immersion time affect the physicochemical characteristics, ELISA-detected BMP-2 levels, degradation behavior, and osteoblast response of hDTM. Overall, lower HCl concentration preserved collagen-like surface features and supported favorable osteoblast proliferation, whereas high HCl concentration damaged the surface microstructure and degraded organic components, leading to increased degradation rates and reduced osteoblast proliferation. Among the tested protocols, 0.5M HCl for 20 minutes provided a practical balance of matrix exposure, controlled degradation, and cellular response.

Preparation of demineralized tooth matrix using HCl directly influenced surface microstructure. SEM analysis revealed that the longer immersion time enlarged dentinal tubule size and exposed collagen fibrils. This observation is consistent with previous report [25] showing that prolonged demineralization increases dentinal tubule diameter and exposes collagen fibers. Enlarged tubules and exposed collagen enhance the effective porous volume, thereby increasing body fluid contact surface area and growth factor release [17, 26]. Interestingly, recent in vitro characterization studies have also reported comparable surface characteristics and elemental composition of processed tooth graft materials relative to conventional allograft materials [1].

Increasing demineralization time led to a decrease in crystallinity percentage, consistent with previous findings [25] showing that the teeth lose their highly crystalline HA structure and become amorphous after 10–30 minutes of acid treatment. XRF analysis also demonstrated reduced calcium and phosphorous content in demineralized groups, consistent with previous reports that demineralization decreases inorganic material content [1, 25, 27], while increasing the relative proportion of organic components.

The Ca/P ratio for stoichiometric HA is approximately 1.67, and this value is often used as a reference for bone mineral [28, 29]. In the present study, all demineralized groups exhibited elevated Ca/P ratios (1.968–2.477), consistent with Ca-rich, non-stoichiometric apatite. However, XRD identified only apatite crystalline phases in all samples, suggesting that the apatite lattice remained detectable after demineralization [9, 30]. This apparent discrepancy can be explained by: (1) differential phosphate loss relative to calcium, altering bulk Ca/P while maintaining apatite diffraction peaks [11, 30]; (2) formation of Ca-rich, amorphous or nanocrystalline calcium-phosphate phases below the detection limit of XRD [28, 29]; (3) lattice substitutions (e.g. carbonate or fluoride) that modify bulk stoichiometry without generating a distinct secondary phase [31, 32]; and (4) methodological differences, as bulk element analyses (XRF/ICP) report total Ca and P, whereas XRD reports only crystalline components [33, 34]. Together, these observations suggest that demineralization induced chemical and structural alterations that increased measured Ca/P while preserving apatite-like crystallinity.

BET analysis revealed that surface area decreased with increasing acid concentration and immersion time, contrasting with a previous study [35] that reported increased surface area in demineralized deciduous teeth. The reduction in surface area observed in the present study was supported by SEM images showing smoother surfaces following demineralization. These results suggest that acid treatment may partially degrade the collagen fibril network, producing a more compact CaP-rich scaffold. In contrast, the increase in average pore diameter with prolonged demineralization likely reflects enlargement of dentinal tubules and formation of surface microcracks.

The degradation rate of the bioceramic materials depends on parameters such as calcium-phosphate content, particle size, and porosity. Increased porosity generally enhances fluid contact and accelerates dissolution [36]. The faster degradation observed at higher HCl concentrations (1M/20 min: 23.76% at day 60) compared with lower concentrations (0.5M/20 min: 10.00% at day 60) likely reflects greater disruption of the collagen matrix. Although rapid degradation may facilitate bone remodeling, excessively fast resorption can compromise mechanical stability before sufficient new bone formation. The moderate degradation rate of the 0.5M/20 min protocol may be compatible with the typical bone healing timeframes (3–6 months), suggesting appropriate temporal matching for clinical applications [37].

BMP-2 is an osteogenic signaling molecule within the transforming growth factor-β (TGF-β) superfamily and is frequently investigated as a biologically relevant factor in demineralized matrices. Reported BMP-2 concentrations in demineralized bone matrix (DBM) range from 3.6 ± 1.20 ng/g [23] to 26.7 ± 11.4 ng/g [38]. In comparison, human deciduous teeth contain approximately 0.42 ± 0.3 ng/g BMP-2, increasing to 1.2 ± 0.3 ng/g after demineralization [39]. The BMP-2 content observed in hDTM in the present study aligns within this range. In this study, BMP-2 levels were quantified by ELISA, reflecting immunoreactive BMP-2 detected in extracted samples. Notably, longer immersion time increased ELISA-detected BMP-2 levels, particularly in the 0.5M/20 min group [15].

The osteoblast response observed in this study likely reflects a combination of surface microstructure, collagen exposure, and mineral characteristics rather than growth factor content alone. Scaffolds incorporating hDTM treated with 0.5M HCl for 20 minutes demonstrated superior cell adhesion and proliferation, which may be attributable to preserved collagen-like features, favorable surface topography, and controlled degradation that maintains a stable substrate for cell attachment.

Our findings concur with Grawish et al. [21], who reported that moderate demineralization conditions preserved dentin-matrix integrity while achieving adequate mineral removal. However, this present study extends those observations by comparing multiple protocols and demonstrating that 20-minute immersion time supported higher ELISA-detected BMP-2 levels across different acid concentrations. Although many protocols employ 30-minute HCl demineralization (0.6 N) [19, 40, 41], and some studies use 60-minute or longer treatments depending on sample type [26, 42], our data indicate that 0.5M HCl for 20 minutes achieves comparable demineralization while better preserving BMP-2 and collagen. This suggests that protocol optimization can reduce processing time while maintaining material quality.

From a clinical perspective, the 0.5M HCl/20 min protocol offers practical advantages, including a short processing time window, balanced degradation kinetics aligned with bone healing timeframes, preservation of growth factor content, and favorable in vitro performance among the tested conditions. The moderate degradation rate (~10.0% over 60 days in vitro) implies that the material can provide structural support during the critical early healing phase while gradually resorbing to allow bone replacement. Nevertheless, these findings should be interpreted as comparative evidence within the studied parameter combinations, and further validation is needed before clinical translation.

In the present study, each demineralization condition was produced in multiple independent processing batches, and the pooled material was used for physicochemical characterization to obtain sufficient mass for multi-instrument testing and to reduce variability. As this work was intended as an exploratory factorial comparison, conclusions are focused on comparative differences within the tested parameter combinations rather than definitive optimization, and larger-scale validation is warranted.

Several limitations should be acknowledged. First, the in vitro SBF degradation model does not fully replicate the complex enzymatic, cellular, and mechanical environments of in vivo bone remodeling. Second, only two HCl concentrations and two immersion times were evaluated; therefore, the proposed protocol should be interpreted as the best-performing condition among the tested parameters rather than a definitive global optimum. Third, BMP-2 quantification was based on ELISA detection of immunoreactive protein and did not assess functional bioactivity following acid treatment. Fourth, the biological evaluation was limited to MC3T3-E1 cells; confirmation using primary human osteoblasts or co-culture systems would strengthen translational relevance. Finally, this exploratory study used limited replicate numbers, which may reduce statistical power to detect small differences; therefore, conclusions should be restricted to comparative trends within the tested conditions and warrant validation in larger-scale studies.

Future studies should focus on: (1) in vivo evaluation of bone formation and graft integration in defect models, (2) functional assessment of osteogenic signaling (e.g. alkaline phosphatase (ALP) activity, osteogenic gene expression, or BMP-responsive assays) to complement ELISA-based BMP-2 detection, (3) analysis of mechanical behavior under physiological loading, and (4) clinical trials to assess safety and efficacy in human applications.

## Conclusion

This study demonstrates that HCl concentration and immersion time influence the physicochemical characteristics, degradation behavior, ELISA-detected BMP-2 levels, and osteoblast response of hDTM. Among the tested protocols, 0.5M HCl for 20 minutes showed a favorable balance of collagen-like surface features, controlled degradation, and enhanced osteoblast-like cell proliferation. These findings support 0.5M/20 min as a practical processing condition within the tested parameter combinations; however, further validation with larger-scale studies is warranted.

## Data Availability

The data sets used and/or analyzed during the current study are available from the corresponding author upon reasonable request.
